# Strain Elastography Evaluation of Patellar Tendons in Dogs after TPLO/TTA for Cranial Cruciate Ligament Rupture, Qualitative and Semiquantitative Evaluation Compared with Healthy Subjects †

**DOI:** 10.3390/ani14202946

**Published:** 2024-10-12

**Authors:** Francesca Del Signore, Stefania De Dominicis, Camilla Smoglica, Martina Rosto, Andrea De Bonis, Andrea Paolini, Massimo Vignoli

**Affiliations:** Department of Veterinary Medicine, University of Teramo, SP 18, 64100 Teramo, Italy; sdedominicis@unite.it (S.D.D.); csmoglica@unite.it (C.S.); mrosto@unite.it (M.R.); adebonis@unite.it (A.D.B.); mvignoli@unite.it (M.V.)

**Keywords:** patellar tendon, dog, TPLO, TTA, strain elastography

## Abstract

**Simple Summary:**

The patellar tendon is described as an intermediate to soft structure with strain elastography, with an increase in stiffness in cases of cranial cruciate ligament rupture. This work aimed to compare the stiffness of healthy ligaments and ones of dogs who underwent TPLO or TTA and to compare the feasibility of a strain ratio with two different reference tissues (cutis/subcutis and infrapatellar fat pad) to semi-quantitatively assess ligament elasticity. The results show that after TPLO or TTA, the ligaments are characterized by increased stiffness, and the distal portion is significantly softer. The cutis/subcutis experience fewer B-mode abnormalities after TPLO or TTA. These data suggest that after surgery, the tendon may become non-uniformly stiffer. The cutis/subcutis appears to be a more reliable reference tissue than the infrapatellar fat pad.

**Abstract:**

Strain elastography (SE) evaluates tissue stiffness, providing qualitative and semiquantitative evaluation, with a strain ratio (SR) > 1 indicating that the target lesion is stiffer than the reference tissue. The patellar tendon has been described as soft in healthy dogs and hard in dogs with cranial cruciate ligament rupture, but SR usefulness has not been assessed. Dogs were divided into healthy (G1) and at least 1 month after surgery (G2) groups. Thickness was recorded, and a B-mode score of 0–3 was determined based on the abnormality’s severity. SE was qualitatively performed with a score of 1–4, and EI was recorded on the whole ligament and in proximal, intermediate and distal areas; SR was collected with the fat pad and cutis/subcutis. G1 was significantly thinner, with a lower score than G2 and a more elastic pattern. EI for G1 was significantly lower than G2 for the whole tendon and the single portions, and EI for the distal portion was significantly lower than the proximal and intermediate one in G2. SR was <1 in G1 and >1 in G2 for both the fat pad and cutis/subcutis. These data suggest that after surgery, the tendon may become non-uniformly stiffer. The cutis/subcutis appears to be a more reliable reference tissue than the infrapatellar fat pad.

## 1. Introduction

The patellar tendon is the quadriceps femoris tendon that connects the patella to the tibial tuberosity. Although its histological structure is typical of a tendon (86% collagen fiber type I), it is often called a “ligament” since it connects two bony structures, i.e., the patella and the tibial crest. In both dogs and humans, the stifle joint supports body weight, transmits the thrust force towards the hip joint, and shortens the functional length of the pelvic limb. The stifle joint has six degrees of movement across three planes: sagittal (flexion/extension and craniocaudal translation), transverse (extra/intra-tibial rotation, and mid-lateral translation), and frontal (adduction/abduction and ventrodorsal translation) [1,2,3,4].

The cruciate ligaments limit hyperextension, tibial intrarotation, and caudocranial tibial translation. Joint stability depends on the balance between intra-articular and periarticular structures (allowing only sagittal flexion–extension) and the forces on the knee (ground reaction and muscle contraction forces). During weight loading, these forces, due to the caudo-distal inclination of the tibial plateau, create a cranially oriented shear force called the cranial tibial thrust. This thrust increases with the tibial plateau inclination and is counteracted by the cranial cruciate ligament (CrCL) [5].

Cranial cruciate ligament rupture (CrCLr) is a common cause of pelvic limb lameness in the canine population and results in lameness, stifle instability and secondary osteoarthritis [6,7], and tibial plateau leveling osteotomy (TPLO) and tibial tuberosity advancement (TTA) are routinely performed in such cases [8,9,10].

Tendinopathy of the patellar tendon in dogs has often been reported after TPLO and TTA, resulting in tendon thickening mainly involving the distal portion; it generally appears as a self-limiting and asymptomatic condition but in several cases can cause marked lameness and prolonged recovery [10,11,12,13,14,15].

Patellar tendon tendinopathy can be easily detected clinically, but the exact damage evaluation can be challenging.

Ultrasound imaging, in particular, is a rapid and non-invasive technique that allows direct tendon visualization, with the possibility to assess ligament thickening, the disruption of fiber orientation, the presence of hypoechoic or anechoic core lesions and increased echogenicity of peritendinous tissues [13,15,16,17].

Although an ultrasound examination provides qualitative evaluations, it does not enable testing of the mechanical and functional proprieties of the structures examined; therefore, it is not possible to clearly quantify the severity of the lesion [18].

Sonoelastography is an ultrasound-based technique that is able to assess the mechanical properties of tissue. This method is based on the principle that by applying mechanical stress to a target tissue, this will be more or less compressed based on its intrinsic elasticity, which could be modified in the case of underlying disorders [19]. The strain is defined as the change in the shape of a tissue while a stress—a force acting on a unit area—is applied to it; the elasticity of a material describes the tendency of tissue to resume its original shape after being deformed by a stress [20].

Sonoelastography evaluates tissue motion by comparing the echoes before and after tissue compression.

The two most common sonoelastography techniques are strain elastography (SE) and shear wave elastography (SWE). SE is based on the measurement of the grade of strain produced by the operator who executes rhythmic and regular compressions with the probe in the area of interest, which is deformed proportionally to its elasticity. The analysis of the deformed tissue is displayed as a strain map, or elastogram, which allows for a qualitative assessment of the tissue stiffness since the manually applied stress is not quantifiable. A semi-quantitative approach is made possible by comparing the relative grade of compression (expressed as the elasticity index (EI) by obtaining the Strain Ratio (SR)) between the target tissue and a reference experiencing the same mechanical stress [21].

SWE, on the contrary, employs a force created by the US pulse to generate shear waves that spread faster in harder tissues [17]. This technique provides quantitatively and objectively measurable velocity expressed in m/s, thus reducing the influence of the user’s skill; however, SWE results are influenced by the depth of the structure investigated, thus making the application on very superficial structures challenging [21,22,23].

In human medicine, SE is currently applied to investigate several tendinopathies affecting the Achilles tendon [24,25], shoulder tendons [26], and the patellar tendon [27,28].

One of the technical principles of the application of SR in the musculoskeletal system is that the reference tissue should be at the same depth as the target one; this is often not possible with tendons or ligaments, thus requiring more superficial or deeper tissues as a reference, for example, the infrapatellar fat pad (or Hoffa’s pad in human medicine) for the patellar tendon [21,27].

The application of SE in the musculoskeletal system may be challenging in the case of curved structures, thus potentially causing artifacts showing increased lateral stiffness due to uneven compression. This can be avoided by using linear probes and maintaining as much contact as possible in the longitudinal plane [29].

Moreover, in the case of cystic lesions, black holes or characteristic “blue–green–red (BGR)” artifacts may be observed; these artifacts, indeed, are useful in confirming the cystic nature of lesions [29].

Furthermore, attention must be paid to the presence of fluid, since it attenuates the propagation of the external stimulus applied at the skin surface, thus impairing accurate measurements [21].

Specifically regarding the patellar tendon, in human medicine, healthy tendons are qualitatively described as soft/intermediate [30], and some concerns about the reliability of SR as a feasible tool to evaluate tendon elasticity have been raised [27,31].

The reproducibility of SR for reference tissues, such as subcutaneous fat and Hoffa’s pad, has been considered poor [23], and in addition, the choice between subcutaneous fat and Hoffa’s pad could lead to a more confusing data interpretation with the lack of standardization [31].

In veterinary medicine, reports are available only for the patellar of healthy subjects, for which the tendons are described as predominantly soft [32,33] with a progressive increase in stiffness in the case of CrCLr [34].

A request to the authors’ facility for stifle USs on canine patients who previously underwent surgery for CrCLr and experienced worsening of clinical conditions without abnormalities detected in post-op X-rays led the authors to investigate the elastographic features of patellar tendons of such patients based on data published by Pennasilisco and colleagues [34].

For such reasons, the authors conceived this work with a dual purpose: first, to compare qualitative elastographic tendon features in healthy and post-surgery dogs to assess eventual elasticity change, and second, to investigate the feasibility of a semiquantitative evaluation of tendon elasticity compared with two different tissues based on the current literature on humans.

## 2. Materials and Methods

Dogs were prospectively recruited and divided into two groups, healthy subjects (G1) and dogs who underwent TPLO or TTA surgery at least a month before the enrollment (G2) from 2021 to 2023; all the dogs included received positive feedback from the surgeon regarding the surgical procedure outcome.

Dogs were classified as healthy after a complete physical and orthopedic examination, with hematology and serum biochemical analysis performed up to six months before enrollment.

For each dog, standard ultrasound (B-mode) and strain elastography (SE) examinations were performed using the Logiq S8 imaging device (GE Healthcare; Milwaukee, WI, USA) equipped with a multifrequency linear transducer (L11, 8.5–10 MHz GE Healthcare; Milwaukee, WI, USA) in association with proper strain elastography software (Logiq S8 Strain Elastography software, GE Healthcare, Milwaukee, WI, USA).

The region was shaved, and a coupling gel was applied; the exam was performed with the patient in lateral recumbency, with the stifle kept in maximal passive flexion to avoid the anisotropy of the fibrillar structures of the ligament [29,31].

Tendon thickness, echogenicity and fiber architecture were recorded and scored as follows:

0: Normal thickness, shape, echogenicity, and fiber architecture;

1: Tendon thickening with normal echogenicity and fiber architecture;

2: Tendon thickening with fiber disruption, changes in echogenicity, and peritendinous hyperechogenicity;

3: Tendon thickening with distinct anechoic core lesions (i.e., complete absence of fiber echos), surrounding fiber disruption and peritendinous hyperechogenicity or edema [15].

Only tendons with a score of 0 were included in the healthy group, whereas tendons showing mineralization, a disrupted pattern, and an increased cross-sectional diameter not related to previous stifle surgery were excluded.

Elastic properties were visualized on the elastogram as a color-coded spectrum ranging from blue to green to red, designating several degrees of tissue elasticity. Low strain, corresponding to less deformable and stiff tissue, was displayed in blue, and high strain, corresponding to more deformable and elastic tissue, was displayed in red.

A dual-screen mode was used to simultaneously display both the B-mode and the elastograms.

Only images without artifacts were evaluated and considered diagnostic when the real-time visual indicator provided by the software determined an adequate degree of correlation of the relative hardness over time (green coil).

According to the elasticity pattern, tendons were evaluated in 4 groups, i.e., blue (hardest tissue; type 1), blue-green (hard tissue; type 2), green (intermediate tissue; type 3), and yellow-red (soft tissue; type 4) [25,30].

The EI was collected on each tendon both on the whole surface (excluding patellar and tibial insertion [32] and on the proximal, intermediate and distal portions.

**Figure 1:**
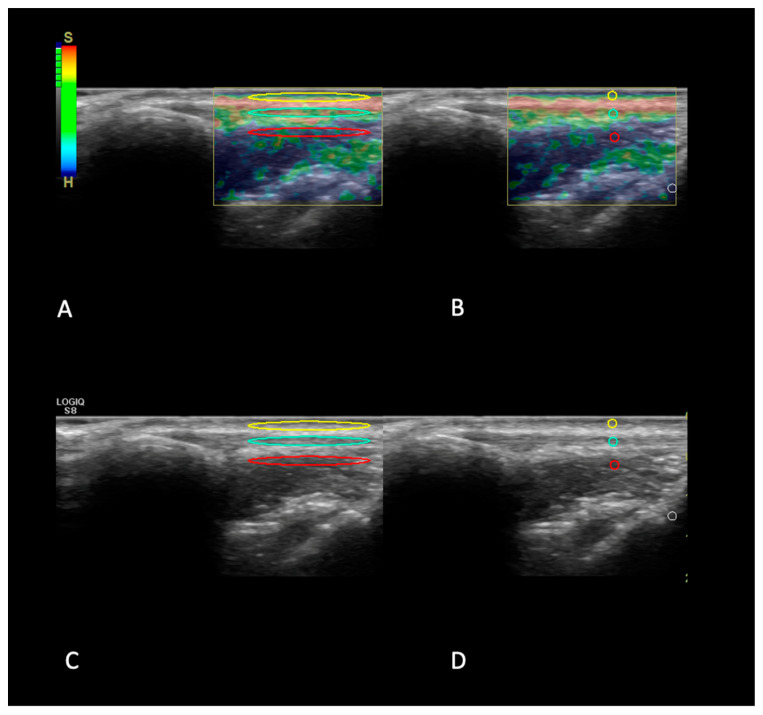
Example of SR measurements. In this picture, an example of the procedure used to measure the SR, with both the cutis/subcutis dorsal to the patellar tendon and the infrapatellar fat ventral to the tendon, is highlighted. In panels (**A**,**B**), the color-coded map (elastogram) is superimposed on the anatomical tendon and on the reference tissues, where differently colored figures highlight the different ROIs on each structure. In panel (**A**), the blue oval highlights the entire tendon surface, while in panel (**B**), the blue circle highlights the central portion of the tendon; the yellow and red circles highlight, respectively, cutis/subcutis and infrapatellar fat pad. The superimposition of the ROIs on the elastogram in panels (**A**,**B**) is the result of the transposition of the ROIs firstly highlighted on B-mode images (panels (**C**,**D**)) on the respective area covered by the elastogram in order to obtain the semi-quantitative values.

**Figure 2:**
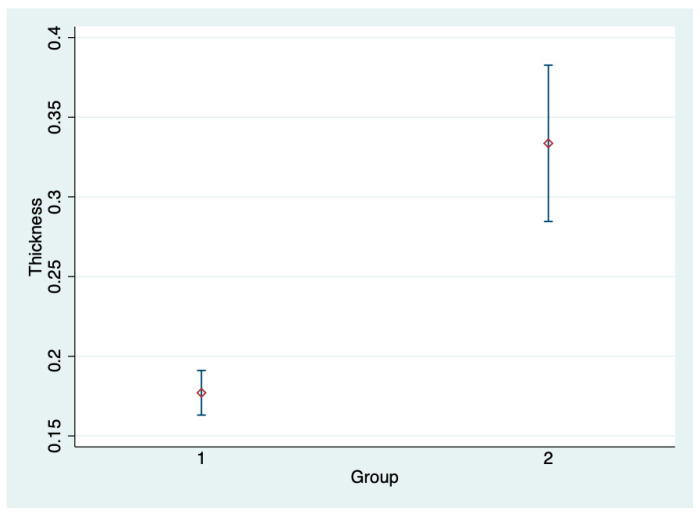
Mean and 95% confidence interval of tendon thickness for G1 and G2.

With the strain ratio calculation, a semi-quantitative result was obtained by comparing tendons with both underlying adipose tissue pad (AT) and underlying cutis/subcutis (CS), following the same protocol for EI measurements (Figure 1 and Figure 2).

The semiquantitative SR evaluations were computed with the use of E-RATIO FUNCTION (GE Healthcare); a value of SR > 1 represented increased tissue stiffness relative to the healthy reference tissue selected.

Statistical analysis was performed with the software package STATA (version Release 17) [35]. Continuous variables were summarized as mean, standard deviation and 95% confidence interval. The Shapiro–Wilk test was performed to evaluate the normal distribution, and the variables not normally distributed were subsequently normalized by transformation. The parametric tests (*t*-test and ANOVA) were performed to compare different groups. Categorical variables were compared using Fisher’s exact test and were described as N and a percentage. The Receiving Operative Characteristic (ROC) analysis was performed, and the area under the curve (AUC) was calculated to assess the accuracy of the SR collected in different portions and with the two different reference tissues. A *p*-value < 0.05 was considered statistically significant.

## 3. Results

### 3.1. Patients and B-Mode US

A total of 24 tendons in G1 and 11 tendons in G2 were included.

The mean age of G1 was 2.5 ± 1.2 years and included, respectively, seven mixed-breed dogs, two Labrador Retrievers, one German Shepherd and one Border Collie.

The mean age of G2 was 7 ± 2.1 years and included, respectively, three mixed-breed dogs, one American Staffordshire, one Cane Corso, one Bovaro of Bernese, one Golden Retriever, one Pitbull and one Labrador Retriever; of these patients, six (66%) experienced pain and lameness at the time of the US.

Of G2, two dogs underwent unilateral TTA, two dogs underwent bilateral TPLO, and five dogs underwent unilateral TPLO; none of these dogs experienced loosening of the surgical device, patellar luxation, osteomyelitis, or tibial crest fracture.

The mean time from surgery was 72 ± 28 days. Clinical data before the surgery were not considered since most of the owners’ reports were too vague to be considered statistically.

The dogs experiencing pain and lameness of the affected limbs experienced variable swelling of the stifle, without other abnormalities from general clinical examination.

The mean tendon thickness for G1 was 0.18 ± 0.03 mm, which was significantly different (*p* < 0.05) from the mean G2 tendon thickness of 0.33 ± 0.07 mm (Figure 2).

Notice the thickness difference between the two groups.

The B-mode evaluation of the tendon showed a significant difference between G1 and G2: all the healthy tendons were classified with a score of zero, while after surgery, tendons were mainly classified with a score from one to three (Table 1).

In G2, the difference in stiffness between dogs who experienced TTA and dogs who experienced TPLO could not be computed due to the low number of cases of TTA surgery.

**Figure 3:**
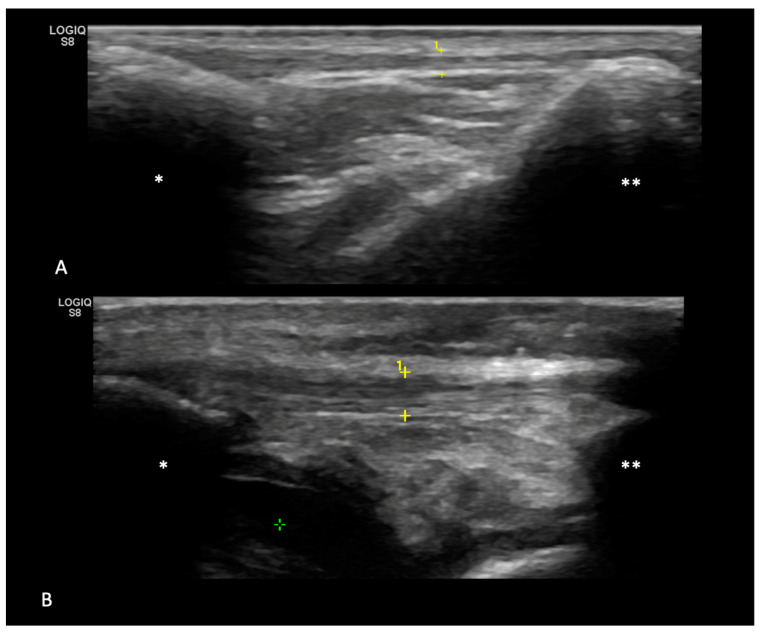
Comparison between a tendon from G1 and a tendon from G2. In panel (**A**), a healthy tendon is highlighted; notice the normal thickness (between yellow calipers), shape, echogenicity, and fiber texture (score 0). Dorsally, cutis and subcutis can be seen, while ventrally, the infrapatellar fat pad is visible. On the left side of the image (*), the distal extremity of the patella can be seen; on the right side of the image, the tibial plateau and tibial crest (**) can be seen. In panel (**B**), a tendon after TPLO is highlighted—notice the increased thickness (between the yellow calipers pointed with numer 1), the partial loss of the fibrillar texture (mainly in the distal portion), with peritendinous fluid and increased echogenicity of infrapatellar fat (Score 3). On the left side of the image (*), the distal extremity of the patella can be seen, and on the right side of the image, the tibial plateau (**) can be seen. The green marker highlights the infrapatellar free fluid.

In Figure 3, an example of the difference in sonographic appearance of tendons from G1 and G2 can be seen.

### 3.2. SE Elastogram Results

**Figure 4:**
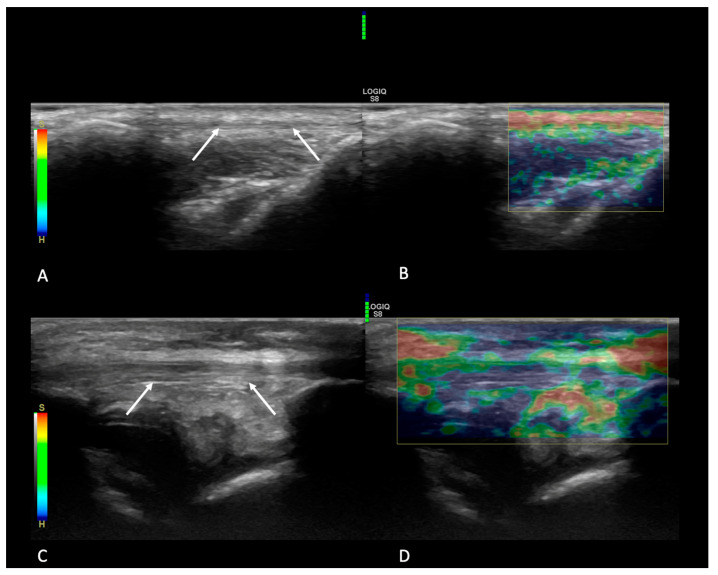
Example of elastogram of two tendons from G1 and G2, respectively. In panels (**A**,**B**), the B-mode image (**A**) and respective elastogram (**B**) of a tendon from G1 are highlighted. The white arrows point to the patellar tendon in panel (**A**), and the yellow-red colors from the elastogram signify that the structure is moderately soft. In panel (**B**), the green-colored bar highlights that the manual compression is correct and that the results from the elastogram can be read. In panels (**C**,**D**), the B-mode image (**C**), and the respective elastogram (**D**), a tendon from G2 is shown. The white arrows point to the patellar tendon in panel (**C**), and the blue-green colors from the elastogram highlight that the structure is hard. In panel (**D**), the green-colored bar shows that the manual compression is correct and that the results from the elastogram can be read.

The qualitative SE evaluation showed a different elasticity between the two groups, with G1 tendons classified as intermediate to soft, and G2 ligaments classified as hardest to hard; see Table 2 for the data distribution, as well as Figure 4 as example.

### 3.3. EI and SR Evaluation

For G1, the mean EI for the entire tendon and for the proximal, intermediate, and distal portions were, respectively, 0.97 ± 0.22, 1.10 ± 0.04, 0.98± 0.17, and 0.94 ± 0.27; no significant difference (*p* < 0.05) was observed between the whole surface and the single portions.

For G2, the mean EI for the entire tendon and for the proximal, intermediate, and distal portions were, respectively, 3.73 ± 0.53, 3.9 ± 1.5, 3.85 ± 0.75, and 2.9 ± 0.91; the distal portion was slightly significantly less stiff (*p* = 0.043) than the total, proximal, and intermediate portions.

The EI of G2 was significantly higher (*p* < 0.05) than in G1 in all the portions examined.

For G1, the SRs of the AT were 0.35 ± 0.13, 0.35 ± 0.14, 0.43 ± 0.17, and 0.5 ± 0.21, respectively, for whole the tendon and the proximal, intermediate, and distal portions; no significant difference was observed between the various portions (*p* > 0.05).

For G2, the SRs of the AT were 1.48 ± 0.67, 1.49 ± 0.77, 1.6 ± 0.6, and 1.9 ± 1.2, respectively, for whole the tendon and the proximal, intermediate, and distal portions; no significant difference was observed between the various portions (*p* > 0.05), but all the portions expressed significantly higher values than in G1 (<0.05).

For G1, the SRs of the CS were 0.93 ± 0.31, 0.74 ± 0.13, 0.75 ± 0.14, and 0.84 ± 0.1, respectively, for whole the tendon and the proximal, intermediate, and distal portions; no significant difference was observed between the various portions (*p* > 0.05).

For G2, the SRs of the CS were 3.6 ± 1.09, 3.3 ± 2, 3.30 ± 0.13, and 3.31 ± 1.81, respectively, for whole the tendon and the proximal, intermediate, and distal portions; no significant difference was observed between the various portions (*p* > 0.05).

The SRs of both the AT and GS were significantly higher in G2 than G1 (*p* < 0.05).

In both G1 and G2, the SRs of the CS were significantly higher than the SRs of AT.

**Figure 5:**
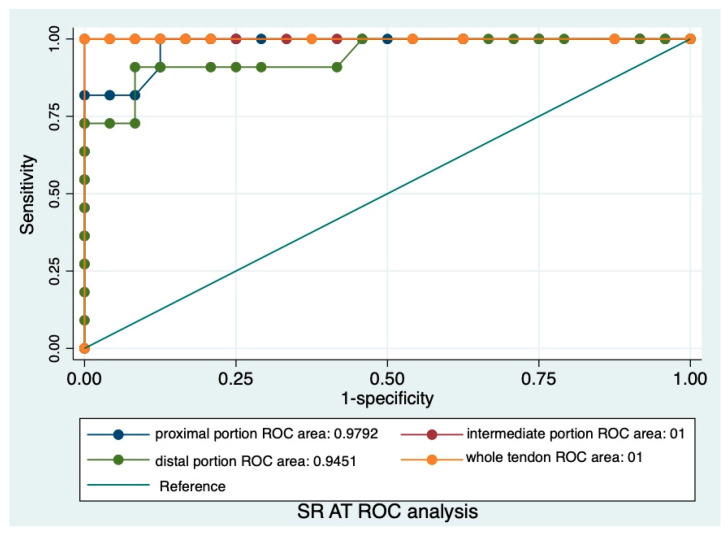
ROC curve analysis of the SR of AT. The area under the curve (AUC) of the SR of AT for proximal, intermediate, and distal portions and the whole tendon.

**Figure 6:**
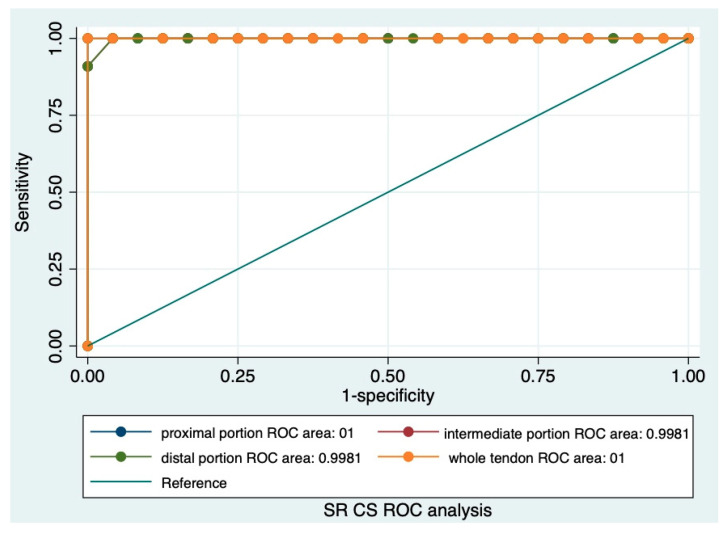
ROC curve analysis of the SR of CS. The area under the curve (AUC) of the SR of CS for proximal, intermediate, and distal portions and the whole tendon.

The ROC analysis showed that the SRs of AT (Figure 5) and CS (Figure 6) are both highly accurate.

## 4. Discussion

This is the first study describing the sonoelastographic features of patellar tendons in dogs affected by CrCLr and treated with TPLO or TTA and the potential role of SRs when using two different reference tissues to semi-quantitatively assess tendon elasticity.

In this work, the authors focused on sonographic and sonoelastographic features of the patellar tendon of dogs who underwent surgical procedures; since a previous report describes that in the case of CrCLr, the tendon experiences an increase in stiffness, the question was raised as to whether this rigidity could also be observed after the surgery, especially in lame and in-pain patients.

A group of healthy dogs was considered as a reference to compare the sonographic and sonoelastographic features.

A qualitative elastogram interpretation clearly shows the difference in the elasticity pattern between healthy and post-surgery tendons, with a more frequent score of 3–4 for healthy tendons and a more frequent score of 1–2 for post-surgery tendons, highlighting that, after surgery, the tendons are characterized by an increase in stiffness compared to a healthy tendon.

The fact that 66% of the dogs included experienced lameness and pain at the time of the US highlights the importance of considering tendon health in surgical planning.

Patellar tendon abnormalities, such as thickening or loss of typical longitudinal fiber patterns, are described as a possible consequence of TPLO or TTA surgery, potentially leading to lameness and pain [11,13,15,36].

Indeed, the potential change in knee biomechanics after TPLO surgery, in particular, has been investigated by DeSandre-Robinson et al., who described how the patellar ligament experiences stressful forces as TPLO reduces the stifle extensor mechanism’s moment arm, meaning that a greater force is required in the patellar ligament to achieve the same torque [36].

While TPLO can eliminate cranial tibial subluxation, it may unintentionally alter the patellofemoral joint mechanics, leading to complications from increased stress on the patellar tendon [37].

Postoperative reduced tibial plateau angles (<6°) are linked to patellar tendinopathy, which involves increased thickness of the distal patellar tendon.

Other factors include changes in patellar tilt or cranial displacement of the tibial tuberosity due to tibial plateau rotation [15].

Moreover, it has been observed that the cranial shifting of the patella relative to the trochlear groove after TPLO alters the patellofemoral kinematics [37].

The increase in stiffness of the patellar tendon observed with SE has been described by Pennasilisco et al. in patellar tendons of dogs affected by CrCLr before surgical intervention.

Pennasilisco and colleagues, indeed, not only observed that there is a significant difference between healthy and “diseased” tendons but also that the increase in stiffness is progressive with increased time of disease onset considering three time intervals from the onset of the lameness, e.g., <15 days, 15 < 60 days and >60 days, thus concluding that evaluating tendon elasticity may be helpful in timing treatment and providing a possible correlation between the onset of postoperative tendinopathy and the condition of the tendon before surgery [34].

The fact that most of the dogs included in this study experienced lameness and sonographic abnormalities after surgery corroborates the usefulness of including sonographic and sonoelastographic evaluation as part of the diagnostic workup in the case of CrCLr in order to fully characterize eventual pre-existing tendinopathies and properly plan the surgical treatment.

The lack of pre-surgery SE data of the dogs included in this study does not allow for an assessment of whether the reduction in elasticity was a pre-existing condition or a consequence of the surgery; however, the fact that the patellar tendon also expresses low elasticity after surgery highlights the benefits of including a stifle ultrasound and patellar tendon SE as important diagnostic workups to manage CrCLr and to fully evaluate tendon health before and after surgery.

In clinical practice, the clinical relevance of patellar tendon thickening observed after TPLO or TTA is still under debate [36].

Most of the dogs included in this study experienced lameness and pain at the time of US examination; however, the size of the samples does not allow for a representative evaluation. It is important to consider that most of the dogs were referred for a second opinion, and it is not possible to perform a representative statistical analysis from the prevalence of these symptoms in all dogs who underwent TPLO or TTA and in each structure where surgeries were performed.

Furthermore, not all patients with a stiff patellar tendon experience lameness after surgery, and this particular aspect should be correlated with the elasticity of the tendon before the surgery. For that purpose, including a complete elastographic evaluation before and after surgery could provide a more valuable evaluation to plan proper treatment.

On the other hand, the fact that the tendons experience a modification in their inner elasticity in cases of CrCLr cannot be ignored, and this finding can also be observed in post-operative evaluation, especially in cases of recrudescence of lameness. For this reason, assessing the elasticity pattern before and after the surgery could improve the post-operative medical treatment or support the inclusion of a preventive physiotherapy protocol in cases of pre-existing high ligament stiffness.

The physiotherapy goals include managing pain, restoring joint movement, strengthening peri-articular and core muscles, correcting proprioceptive deficits, and preventing prolonged limb disuse to avoid muscle atrophy, reduced joint mobility, cartilage loss, weakened tendons and ligaments, and osteopenia [5]. In cases of increased stiffness of the patellar tendon detected before surgery, a treatment plan including physiotherapy before and after surgery could be useful to reduce progressive tendinopathy and the related clinical symptoms.

Regarding the semiquantitative approach, in this work, EIs of the tendons were collected from both the whole tendon surface and the single tendon portions, i.e., proximal, intermediate, and distal. The tendon length analyzed was decided based on previously published data [32,33].

It was observed that in healthy tendons, the elasticity is uniform, without statistically significant differences in values in the various portions; this is consistent with previously published data [32].

A slight difference regarding EI values was observed in the tendons of dogs who underwent surgery, with a reduction in the EI in the distal portions; this difference, even if slight, may signify that the elasticity change in tendons may not be uniform.

These data are not surprising since some dogs experience a selective thickening of the distal portion of the tendon [35], and the non-uniform tendon thickening may correlate with a non-uniform loss of elasticity. The lack of histopathological data limits the correct interpretation of these findings.

Finally, to obtain the SR, two different reference tissues were considered, i.e., the infrapatellar fat pad and cutis/subcutis. The choice of such reference tissues was motivated by purely anatomical reasons and by the available literature on human medicine [27,31].

Overall, the healthy tendons expressed an SR < 1 or close to 1 for both AT and CS, while the diseased tendons expressed significantly higher SRs for both reference tissues; these data are perfectly in line with the increase in stiffness of the tendons in dogs after surgery.

The ROC curve shows that both SRs can accurately distinguish between soft and hard tendons; however, a technical clarification is mandatory.

The rationale for choosing tissue as a reference to obtain the SRs is that the tissue is healthy and thus can be considered “standard” for comparing the effect of the same mechanical stress on both healthy and diseased target tissue, leading to reproducible results [21].

This is not always achievable in cases of articular disorders, where the presence of articular fluid and inflammation inevitably also alters the reference tissues, such as the infrapatellar fat pad; furthermore, the fluid movement does not reflect the stiffness of the solid tissues, thus impairing the SE results based on mechanical tissue compression [21].

For such reasons, care needs to be taken in the choice of the reference tissues used to obtain the SR. The authors suggest considering the CS as the first reference tissue in cases of B-mode sonography abnormalities involving the articular space.

In human medicine, indeed, the available reports failed to standardize absolute SR reference values for both subcutaneous fat tissue and the infrapatellar fat pad, thus questioning the real usefulness of these parameters and suggesting the use of a standard gel pad as a reference [27,31].

The authors partially agree with this point and, based on the data presented in this study, invite clinicians to not consider SRs as absolute values. Instead, they suggest first to perform a qualitative elastogram evaluation and then to intend the SR in terms of < or >1 to assess the eventual increase or decrease in tendon elasticity, without placing excessive focus on the exact value for absolute reference intervals.

The major drawback of this study is the lack of pre-surgery US and SE data. This is mainly because most of the tendons included were scanned for a second opinion before starting a physiotherapy protocol after recrudescence of lameness after surgery without evidence of surgical device problems. The fact that the dogs were scanned only after the surgery for a sonographic evaluation allowed only a partial evaluation of the stiffness variation in the tendon before and after surgery. The data published by colleagues regarding an increase in tendon stiffness after CrCLr support the idea that evaluating tendon stiffness before and after surgery could be helpful in the clinical field to properly plan the treatment of such patients.

The second limit is based on the comparison of non-homogeneous groups, either in terms of the number of subjects or in terms of the homogeneity of the subjects regarding breed, weight, and age. The latter aspects, in particular, may have played an important role both in the development of CrCLr and in the change in tendon stiffness; it is well known, indeed, that this condition has a multifactorial etiology, and the increase in age of the post-op patients could have partially increased the overall stiffness of the tendon.

The lack of a standardized stiffness evaluation in healthy patients of different ages makes absolute comparisons difficult and highlights the importance of future studies to standardize the procedure.

Also, it was not possible to standardize the sonographic examination based on the time between the surgery and the ultrasound since most of the patients experienced lameness at different times after surgery.

Indeed, performing a US after the surgery without a complete imaging record both pre- and post-surgery may limit several considerations regarding stifle joints. For this reason, including sonoelastography of the patellar tendon in the diagnostic workup before and after TPLO/TTA surgery could provide a more detailed understanding of the role of tendon elasticity changes at the patient’s follow-up, especially for patients experiencing lameness in the absence of surgical device complications.

Specifically regarding the reference tissue used to obtain the SRs, the authors did not compare SRs obtained with a reference material (such as a gel pad), but in the future, it could be useful to evaluate such options to avoid bias in the measurements.

Finally, the lack of more advanced techniques used as a control (such as MRI) to confirm tissue abnormalities may limit a thorough understanding of underlying structural changes leading to elasticity modification.

## 5. Conclusions

SE is a rapid and non-invasive diagnostic tool for assessing increased stiffness in the patellar tendon of dogs. Qualitative evaluation provides useful information for comparing healthy and diseased tendons, with an SR > 1 indicating increased stiffness when considering both AT and CS as references. However, care should be taken to check for healthy reference tissue in the case of tendon disease.

The data presented herein, even with their limitations, support the use of sonoelastography to evaluate patellar tendon health after surgical procedures and highlight that including this examination before and after surgery could improve diagnostic accuracy and provide important information in surgical planning and follow-up.

## Figures and Tables

**Table 1:** B-mode score distribution between G1 and G2 ligaments.

B-Mode Score	Freq. (Percent)		
	Group		
	G1	G2	Fisher’s Exact Test
0	24/24 (100%)	1/11 (19%)	*p* < 0.000
1	0/0	3/11 (27%)	
2	0/0	2/11 (18%)	
3	0/0	5/11 (46%)	

**Table 2:** SE qualitative score distribution between G1 and G2 tendons.

SE Qualitative Evaluation	Freq. (Percent)		
	Group		
	G1	G2	Fisher’s Exact Test
1	0/24	5/11 (45.5%)	*p* < 0.000
2	0/24	6/11 (54.5%)	
3	18/24 (75%)	0/11	
4	6/24 (25%)	0/11	

## Data Availability

All the data are provided within the manuscript.
